# Central nervous system relapse after allogeneic HCT in FLT3-mutated AML

**DOI:** 10.1007/s00277-024-06106-y

**Published:** 2024-11-26

**Authors:** Khouloud Kouidri, Fabian Acker, Rosa Toenges, Saskia Pfaff, Sarah Lindner, Julia Riemann, Marek Werth, Salem Ajib, Fabian Lang, Björn Steffen, Thomas Oellerich, Hubert Serve, Anjali Cremer, Gesine Bug

**Affiliations:** 1 Department of Medicine 2, Hematology/Oncology, Goethe University Frankfurt, University Hospital, Frankfurt, Germany; 2 Department of Hematology, Oncology and Cancer Immunology, Charité - Universitätsmedizin Berlin, corporate member of Freie Universität Berlin and Humboldt-Universität zu Berlin, Berlin, Germany; 3 Dana Farber Cancer Institute, Boston, MA United States of America

**Keywords:** FLT3, AML, HCT, CNS, Relapse, Real-World Data, Retrospective

## Abstract

Central nervous system (CNS) relapse in acute myeloid leukemia (AML) is rare, but prognostically extremely unfavorable and associated with very high mortality rates. Aim of our single-center study was to define risk factors for CNS relapse in patients with *FLT3*-mutated AML after allogeneic hematopoietic cell transplantation (HCT) and to determine the impact of pre-emptive or salvage therapy with FLT3-inhibitors (FLT3i) on occurrence of CNS relapse and overall prognosis. We analyzed 39 *FLT3*-mutated AML patients who were treated with intensive induction therapy and consecutively underwent HCT at our institution. We observed that the remission status before HCT was strongly associated with relapse probability. Notably, *FLT3*-mutated AML showed a high propensity for CNS relapse with a cumulative incidence of 50% (95% confidence interval [CI], 16–77) at 2 years in non-responders pre-HCT compared to 0% in responders (cause-specific hazard ratio, 24.5, 95% CI, 2.9-206.2; *p* = 0.003). This was not abrogated by pre-emptive or salvage therapy with FLT3i in first relapse. Targeted therapies prior to transplant, use of intrathecal chemoprophylaxis or closer monitoring may be considered in patients with FLT3-mutated AML with active disease prior to HCT in order to prevent CNS relapse.

**Supplementary Information:**

The online version contains supplementary material available at 10.1007/s00277-024-06106-y.

## Introduction

Mutations in the FMS-like tyrosine kinase 3 (*FLT3*) gene are present in nearly 30% of acute myeloid leukemia (AML) cases [1]. The increased relapse risk and unfavorable outcomes associated with *FLT3* internal tandem duplications (ITD) may be counteracted by treatment with specific FLT3 inhibitors (FLT3i) and allogeneic hematopoietic cell transplantation (HCT) [2]. In contrast, mutations in the *FLT3* tyrosine kinase domain (TKD) do not appear to negatively influence the prognosis [3]. The discovery of *FLT3* mutations and their prognostic significance led to the development of numerous targeting compounds: both midostaurin and quizartinib have been shown to improve overall survival (OS) in combination with standard chemotherapy-based induction and consolidation therapy [4, 8]. Sorafenib is recommended as maintenance therapy post-HCT irrespective of measurable residual disease (MRD) prior to HCT [5, 6]. The second-generation FLT3i gilteritinib was approved as monotherapy for the setting of relapsed or refractory *FLT3*-mutated AML [7]. Overall, FLT3i have significantly impacted AML treatment, improving overall survival rates in various clinical scenarios.

However, the efficacy of FLT3i in extramedullary disease including central nervous system (CNS) involvement remains unclear. In general, CNS involvement in AML is rare and associated with an increased risk of morbidity and mortality with a 5-year OS of only 11% [9]. The exact incidence of CNS involvement in adult patients with AML is unknown, as routine cerebrospinal fluid (CSF) sampling is rarely performed in neurologically asymptomatic patients. Risk factors for CNS involvement in AML identified so far include high white blood cell count (WBC) and elevated lactate dehydrogenase (LDH) levels at first diagnosis [9, 10], but little is known about risk factors for CNS relapse after HCT. Aim of this retrospective single center study was to evaluate the frequency of CNS relapse in patients with *FLT3*-mutated AML who underwent HCT, to determine underlying risk factors and to analyze the impact of FLT3-targeted therapies.

## Methods and study design

Inclusion criteria for this retrospective study were adult patients with *FLT3*-mutated AML (*FLT3*-ITD and/or *FLT3*-TKD) and first HCT between January 2017 and December 2020 at our transplant center at Goethe University Hospital Frankfurt, Germany. Primary endpoint of the study was the cumulative incidence of clinically apparent CNS relapse. Of note, no routine screening for CNS involvement is implemented at our department and, thus, only symptomatic patients received lumbar puncture and/or imaging of the head and spine.

Clinical data including conditioning regimens and pre-emptive and maintenance therapy with FLT3i were retrospectively retrieved from electronic health records. Conditioning regimens for HCT were defined as myeloablative (MAC), non-myeloablative (NMA) or reduced intensity (RIC) according to Bacigalupo et al. and Giralt et al. [11, 12]. Treatment response was defined according to the European Leukemia Network (ELN) 2017 criteria as bone marrow blasts < 5% in the absence of circulating blasts and extramedullary disease, either with complete (CR) or incomplete (CRi) hematologic recovery (absolute neutrophil count [ANC] ≥ 1.0 × 10^9^/L, platelet count ≥ 100 × 10^9^/L) [2]. Measurable residual disease (MRD), was determined prior to and days after HCT by RT-qPCR (NPM1mut, *n* = 26; KMT2A-PTD, *n* = 2; RUNX1::RUNX1T1, *n* = 2; and RUNX1mut, NUP98::NSD1, *n* = 1 each) or multiparameter flow cytometry (*n* = 7).

All data obtained in this study involving human participants were collected in accordance with ethical standards of our institutional research committee and with the 1964 Helsinki Declaration. Written informed consent was obtained from all patients and the study was approved by the Ethical Committee at the University Hospital Frankfurt (project-number: SHN-1-2022).

Statistical analysis was performed using R version 4.3.3. The hazard ratio (HR) for CNS relapse was calculated using a cause-specific hazard model considering all-cause death a competing risk. Progression-free survival (PFS) and OS were calculated using Cox proportional hazard models and compared using a log-rank test.

## Results

**Fig. 1:**
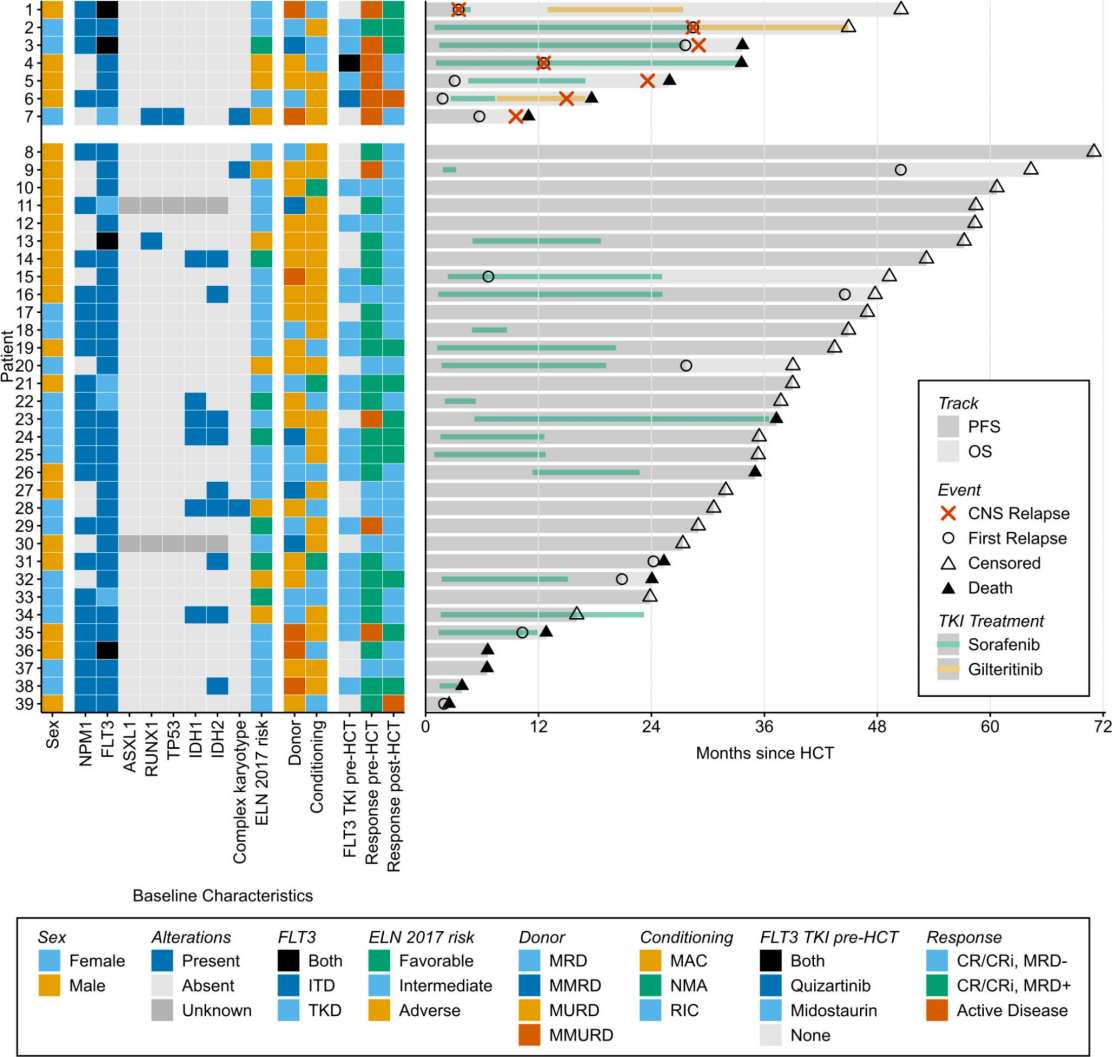
Baseline characteristics and swimmer plot

We screened 273 patients who underwent the first HCT at our institution between January 2017 and December 2020 and retrospectively analyzed the data of 39 patients with *FLT3*-mutated AML who received intensive induction prior to transplant. Patient and treatment characteristics are detailed in Table 1. Of the 39 patients harboring a *FLT3* mutation, 23 patients (58%) received a FLT3i at some time during their treatment prior to transplant, mainly midostaurin (Fig. 1). No patient had CNS involvement prior HCT and no prophylactic intracranial chemotherapy was given during induction.

**Table 1:** Overview of analyzed patients with FLT3-ITD (*n* = 34) and/or TKD-mutated AML (*n* = 5) who were transplanted between 2017 and 2020 at our institution. (7 + 3: cytarabine given as a continuous intravenous infusion over 7 days and daunorubicin given as an intravenous infusion over 3 days. HAM: high-dose cytarabine and mitoxantrone, GVHD indicates graft-versus-host disease)

Patient characteristics (*N* = 39)	*N* (%)
Age – median (range)	53 (20–75)
**Sex**	
male female	22 (56) 17 (44)
**Mutations**	
FLT3-ITD / NPM1mut FLT3-ITD / NPM1wt FLT3-TKD	22 (56) 12 (30) 5 (12)
**Genetic Risk Group (ELN2017)**	
favorable intermediate adverse	8 (21) 23 (59) 8 (21)
**Genetic Risk Group (ELN2022)**	
favorable intermediate adverse n/a	3 (8) 27 (69) 7 (18) 2 (5)
**Induction therapy**	
7 + 3 7 + 3, followed by HAM 7 + 3, followed by azacitidine-venetoclax	23 (59) 15 (38) 1 (3)
**Donor type**	
matched-related haploidentical matched-unrelated mismatched-unrelated	10 (26) 5 (13) 18 (46) 6 (15)
**Conditioning regime**	
myeloablative Fludarabine + Melphalan + TBI Fludarabine + TBI Fludarabine + Busulfan + Thiotepa Fludarabine + Busulfan Busulfan + Cyclophosphamide non-myeloablative Fludarabine + Melphalan + TBI reduced-intensity Fludarabine + Melphalan + Thiotepa Fludarabine + Melphalan	25 (64) 15 (38) 2 (5) 5 (13) 2 (5) 1 (3) 3 (8) 3 (8) 11 (28) 3 (8) 8 (21)
**GvHD prophylaxis**	
CSA-MMF-ATG CSA-MMF CSA-MMF-PTCY CSA-MTX-ATG Tac-MMF-PTCY Tac-MMF-ATG Tac-MMF	22 (56) 5 (13) 4 (10) 3 (8) 3 (8) 1 (3) 1 (3)
**Remission status pre-HCT**	
CR CR1, MRD negative CR1, MRD positive CR2, MRD negative CR2, MRD positive Active disease	29 (74) 6 (15) 17 (44) 2 (5) 4 (10) 10 (26)
**Remission status post-HCT**	
CR CR1, MRD negative CR1, MRD positive CR2, MRD negative CR2, MRD positive Active disease	37 (95) 21 (54) 9 (23) 5 (13) 2 (5) 2 (5)

At the time of HCT, 8 patients (21%) were MRD-negative, 21 (54%) in MRD-positive CR/CRi and 10 (26%) had active disease. Twenty-six (67%) patients received a myeloablative conditioning regimen. After a median of 64 days post-HCT (range, 53–110), 37 patients (95%) were in CR/CRi with 26 (67%) also having MRD-negative disease. After transplant, sorafenib was initiated prophylactically (*n* = 10) or pre-emptively (*n* = 10) after a median of 50 days (range, 28–346) after HCT for a median duration of 13.5 months (1.4–32.2). Reasons for not receiving FLT3i treatment included HCT before maintenance therapy was routinely used in 2019 (*n* = 12), renal insufficiency (*n* = 1), FLT3-TKD only (*n* = 3), and early relapse (*n* = 2).

**Fig. 2:**
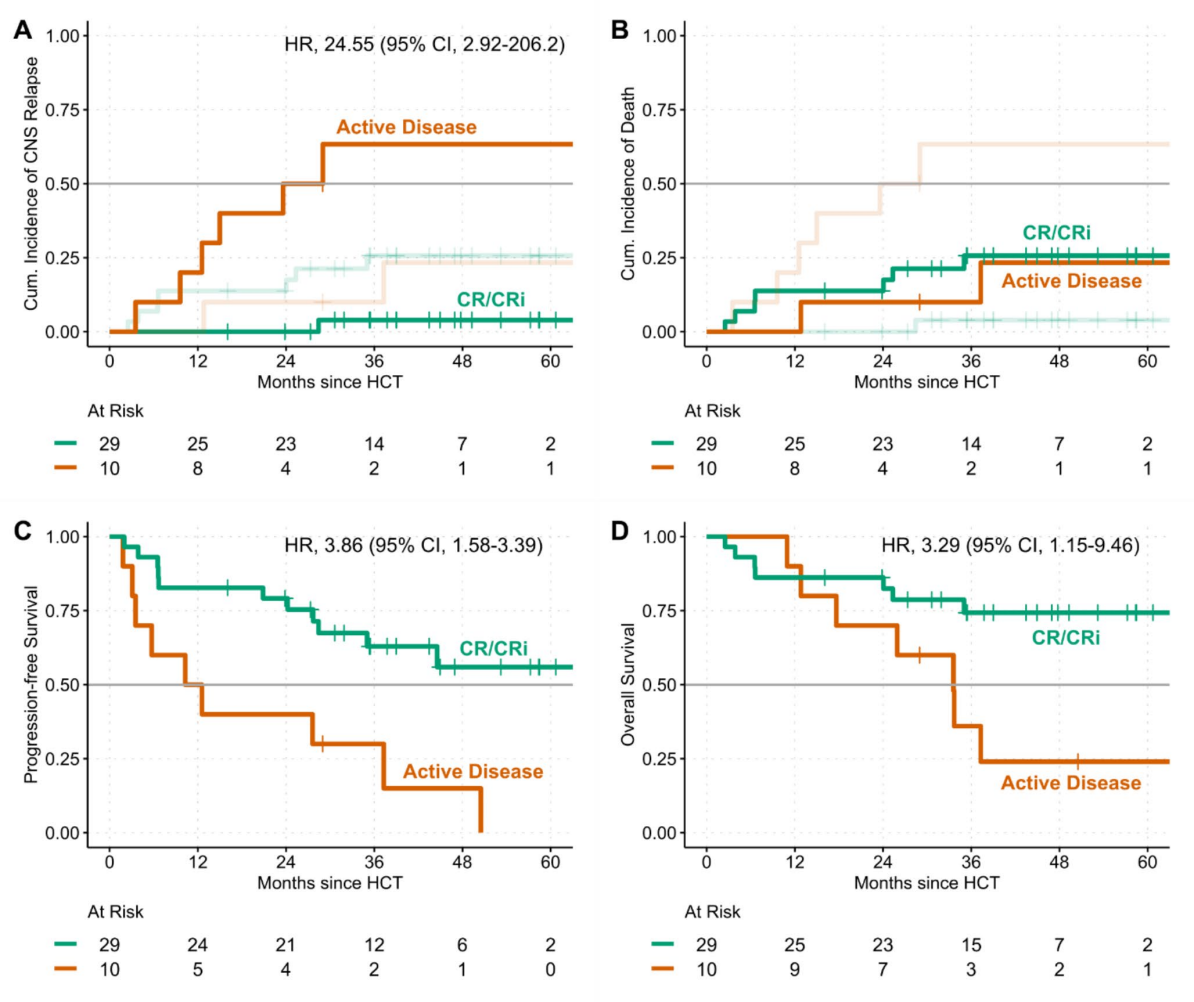
Competing risk analysis and survival outcomes. Panels A and B show the cumulative incidence of CNS relapse (**panel A**) considering all-cause mortality a competing risk (**panel B**). Panels C and D illustrate progression-free (**C**) and overall survival (**D**). CI denotes confidence interval, CR/CRi complete response with/without recovery, Cum. Cumulative, HCT hematopoietic stem cell transplantation, and HR hazard ratio

With a median follow-up of 44.9 months (range, 16.1–71.0), CNS relapse was observed more frequently in patients without CR/CRi pre-HCT (Figs. 1 and 2A-B). After 1 and 2 years, the cumulative incidence of CNS relapse was 20% (95% CI, 3–49) and 50% (95% CI, 16–77) in patients without CR/CRi compared to 0 of 29 (0%) in patients with CR/CRi. The cause-specific hazard ratio for CNS relapse was 24.5 (95% CI, 2.9-206.2; *p* = 0.003). CNS relapse (*n* = 7) occurred both as the first (*n* = 4) and later (*n* = 3) event of disease progression (Fig. 1). All but one patient had positive CSF cytology. Of six patients with brain/spine MRI, two had evidence of parenchymal lesions (33%) including the patient with negative CSF cytology. A landmark analysis of OS from the time of recurrence is shown in Supplemental Fig. 1.

Treatment of CNS relapse consisted of intrathecal chemotherapy (12 mg or 15 mg methotrexate, 40 mg cytarabine and 4 mg dexamethasone) in all six patients with meningeal involvement. The number of administered intrathecal therapies depended on the response and was conducted 5, 6, 9, 10, 19, and 22 times (median, 9.5). Five patients received additional gilteritinib. Radiation therapy was administered in five patients with three receiving whole-brain irradiation and two spinal irradiation. All patients were treated with donor lymphocyte infusions at some point after relapse.

PFS was shorter in patients not achieving CR/CRi prior to HCT with a median PFS of 11.4 months (95% CI, 3.5-not estimable [NE]). Median PFS was not reached in CR/CRi patients. HR for disease progression or death was 3.86 (95% CI, 1.58–9.39; *p* = 0.003; Fig. 2C). Accordingly, OS was shorter in patients without CR/CRi prior HCT with the median OS being 33.6 months (17.6-NE) vs. not reached. HR for death was 3.29 (1.15–9.46; *p* = 0.027; Fig. 2D).

## Discussion and conclusion

CNS involvement of AML may cause significant morbidity and mortality. In our study, patients with FLT3-mutated AML and active disease at the time of HCT had a cumulative incidence of CNS recurrence of 50% at two years. In a large retrospective study by the Acute Leukemia Working Party of the European Society for Blood and Marrow Transplantation, it was demonstrated that despite being a rare event in AML, CNS relapse is associated with a dismal prognosis [20]. Our data suggest that patients without CR/CRi pre-HCT should be monitored closely for neurologic symptoms.

In addition, the treatment of these patients should aim to minimize the risk of CNS relapse. However, it remains unclear whether the risk of CNS relapse may be mitigated by any particular treatment strategy. In the RATIFY and QuANTUM-First trials, midostaurin and quizartinib, respectively, improved relapse-free survival (RFS) and OS compared to placebo when given during induction and chemotherapy consolidation therapy [4, 8]. In the post-transplant setting, sorafenib (SORMAIN trial, NCT02474290) and gilteritinib (MORPHO trial) maintenance therapy were shown to significantly reduce relapse risk [5, 6, 13]. Despite the fact that an intracranial activity of gilteritinib and sorafenib has previously been demonstrated [14, 15, 23, 24] the incidences of CNS recurrence have not been reported for either of the aforementioned trials.

It also remains unclear whether different induction chemotherapies have an impact on post-HCT CNS recurrence. High-dose cytarabine is known to penetrate the blood-brain barrier and reach therapeutic concentrations in the CSF [17] and, thus, is widely used in the treatment of several hematologic malignancies including CNS lymphoma [25] The NCRI AML19 trial randomized patients with newly diagnosed AML between FLAG-Ida plus gemtuzumab-ozogamicin (GO) and CPX-351 with no significant difference in RFS between arms. However, as patients in the FLAG-Ida plus GO arm received cytarabine at CNS-active single doses of 2000 mg/m², a secondary analysis of CNS vs. non-CNS relapse may be interesting [16]. 

In acute lymphoblastic leukemia (ALL), the CNS is the most frequently involved extramedullary site. In a retrospective study comprising 53 ALL patients with CNS disease who underwent HCT using total body irradiation (TBI)-based conditioning regimens, 30 patients received a cranial radiation boost. Of these 30 patients, none had CNS relapse after HCT compared to 3 out of 23 patients without boost (13%) [19]. In our cohort, 20 of 39 patients received TBI albeit no cranial boost was performed. However, due to the small sample size we are unable to make any conclusions regarding the impact of TBI on the incidence of post-HCT CNS relapse. Of note, the long-term effects and potential side effects of additional whole-brain irradiation must be taken into consideration [18]. 

The optimal treatment of CNS disease after HCT is unclear. In analogy to ALL, intrathecal chemotherapy is commonly used in AML [22, 26]. In our study, 6 out of 7 patients with CNS relapse after HCT received intrathecal chemotherapy, either in combination with radiotherapy or with targeted therapy. Two patients with CNS relapse after transplant responded with MRD negative CR to gilteritinib, one in combination with intrathecal chemotherapy and one in combination with irradiation of meningeal chloroma. Although the effect of gilteritinib on CNS disease cannot be deduced from our data as it was combined with other modalities, our findings align with previous case reports or series demonstrating durable CNS responses with gilteritinib [14, 15].

The interpretation of our results is subject to all the limitations inherent to retrospective analyses. As CNS involvement may remain asymptomatic and no routine imaging or CSF sampling was conducted, the actual incidence of CNS relapse may have been underestimated in our study. However, in a post-hoc analysis of multiple prospective AML trials, the incidence of CNS disease in five studies that required lumbar puncture as part of the study protocol was comparable to the incidence observed in studies where lumbal puncture was performed at the investigator’s discretion [21]. Given the small sample size of our study, no formal analysis of the association of genetic co-alterations and the risk of CNS relapse was done. However, no clear correlation with high-risk genetic alterations (ASXL1, TP53, RUNX1) was noticed (Fig. 1).

In conclusion, our data suggest that additional therapy may be considered for patients with *FLT3*-mutated AML and active disease prior to transplant where relapse with CNS involvement seems to be common. Optimizing induction therapy, prophylactic intrathecal therapy, cranial boost during TBI, and closer monitoring may be considered in patients with *FLT3*-mutated AML and no CR/CRi before HCT.

## Electronic supplementary material

Below is the link to the electronic supplementary material.

Supplemental Fig. 1: Landmark analysis showing overall survival from the time of first relapse by the type of relapse (CNS vs. Non-CNS) excluding patients without relapse or death before relapse

## Data Availability

The datasets generated and analyzed in the current study are available from the corresponding author on reasonable request.
