# Supplementary material for: Central nervous system relapse after allogeneic HCT in FLT3-mutated AML
Source: Ann Hematol. 2024 Nov 26;103(12):5387–93. doi: 10.1007/s00277-024-06106-y (PMC11695448; doi:10.1007/s00277-024-06106-y)

**Central nervous system relapse after allogeneic HCT in FLT3-mutated AML**

Khouloud Kouidri^1^*, Fabian Acker^1^*, Rosa Toenges^1,3^,^,^ Saskia Pfaff^1^, Sarah Lindner^1,2^, Julia Riemann^1^, Marek Werth^1^, Salem Ajib^1^, Fabian Lang^1^, Björn Steffen^1^, Thomas Oellerich^1^, Hubert Serve^1^, Anjali Cremer^1^, Gesine Bug^1^

* Authors contributed equally

*Affiliations*

*^1^ Goethe University Frankfurt, University Hospital, Department of Medicine 2, Hematology/Oncology, Frankfurt, Germany*

*^2^ Department of Hematology, Oncology and Cancer Immunology, Charité - Universitätsmedizin Berlin, corporate member of Freie Universität Berlin and Humboldt-Universität zu Berlin, Berlin, Germany*

*^3^ Dana Farber Cancer Institute, Boston, Massachusetts, United States of America*

**Corresponding author:** Khouloud Kouidri; University Hospital Frankfurt, Department of Hematology and Oncology, Theodor-Stern-Kai 7, 60590 Frankfurt am Main, Germany; e‑mail: Kouidri@med.uni-frankfurt.de; phone +49 69 6301 6566; fax +49 69 6301 6567

# **Supplemental Material**

***Supplemental Figure 1 – Landmark analysis showing overall survival from the time of first relapse by the type of relapse (CNS vs. Non-CNS) excluding patients without relapse or death before relapse***


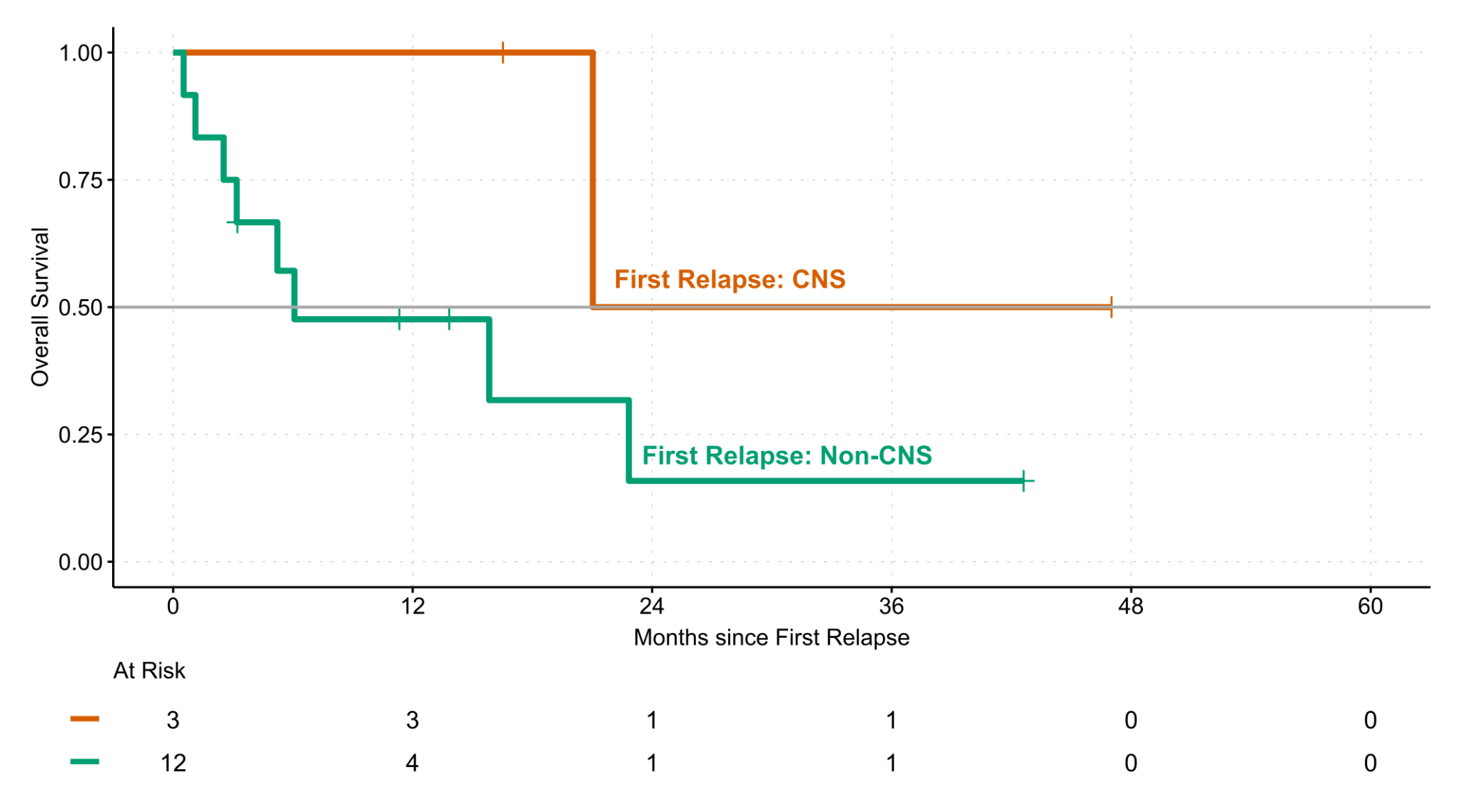

Supplement: Supplementary file 1 — Supplemental Fig. 1: Landmark analysis showing overall survival from the time of first relapse by the type of relapse (CNS vs. Non-CNS) excluding patients without relapse or death before relapse [file 277_2024_6106_MOESM1_ESM.docx]
